# Co-Occurrence of *Fusarium* and *Alternaria* Metabolites in Brewing Barley Monitored during Two Consecutive Years (2019–2020)

**DOI:** 10.3390/biom14091156

**Published:** 2024-09-14

**Authors:** Kristina Habschied, Krešimir Mastanjević, Jurislav Babić, Rudolf Krska, Michael Sulyok, Alojzije Lalić, Gordana Šimić, Tihomir Kovač

**Affiliations:** 1 Faculty of Food Technology Osijek, Josip Juraj Strossmayer University of Osijek, F. Kuhača 20, 31000 Osijek, Croatia; kristinahabschied@gmail.com (K.H.); jbabic@ptfos.hr (J.B.); tkovac@ptfos.hr (T.K.); 2 Department of Agrobiotechnology (IFA-Tulln), Institute of Bioanalytics and Agro-Metabolomics, University of Natural Resources and Life Sciences Vienna, Konrad Lorenzstr. 20, A-3430 Tulln, Austria; rudolf.krska@boku.ac.at (R.K.); michael.sulyok@boku.ac.at (M.S.); 3 Institute for Global Food Security, School of Biological Sciences, Queen’s University Belfast, University Road, Belfast BT7 1NN, UK; 4 Agricultural Institute Osijek, Južno predgrađe 17, 31000 Osijek, Croatia; alojzije.lalic@poljinos.hr (A.L.); gordana.simic@poljinos.hr (G.Š.)

**Keywords:** barley, infectopyron, siccanol, LC-MS, crop protection

## Abstract

Known mycotoxins have been investigated for years. They have been included in legislation and are meticulously controlled in most cereals, cereal-related products, and raw materials of animal origin. However, there are still mycotoxins that need to be addressed by regulations and subsequently are not monitored but can still occur in relatively high concentrations. This research aimed to assess the occurrence of common *Fusarium* mycotoxins in hulled barley. Samples of hulled barley were treated in the field with two protective treatments, alongside a control sample sans treatment. Furthermore, we aimed to assess the occurrence of *Alternaria* mycotoxins in the chosen samples. The results have shown that *Fusarium* mycotoxins were mostly determined by climatic conditions (no mycotoxins in 2020, except siccanol). Most interesting was the appearance of infectopyron, an *Alternaria* toxin that was detected in all samples in 2019 and in the majority of samples in 2020. The highest concentration was detected in 2019 in hulled barley with 536 µg/kg, while in 2020, the highest concentration of this mycotoxin reached 350 µg/kg. These findings depict the need for further research on food safety regarding mycotoxins, and the need for additional changes in legislation. This investigation shows that fungicide application in rainy years cannot efficiently suppress mycotoxin production. Additionally, even in dry years, some of the mycotoxins not involved in legislation, such as infectopyron and siccanol, do not respond to the application of fungicides.

## 1. Introduction

The malting and brewing industry mainly relies on barley (*Hordeum vulgare* L.) as the main cereal. In addition, it is grown globally and still successfully resists the effects of global warming, at least yield-wise. However, abrupt and extreme climate changes significantly impact the microbial diversity of cereals best observed in the mycotoxicological profile. There have been reports on *Fusarium* species shifts, and they are noted globally [1,2,3,4]. Global warming has caused the retreat of *Fusarium culmorum*, a fungus commonly found in Central and Eastern European countries, and has enabled the spreading of *Fusarium graminearum*, a fungus indigenous to warmer climatic areas [1]. An average temperature increase of 2.5–5.0 °C across European countries in combination with precipitation alterations could give rise to higher yields for northern, western, and Atlantic areas, while southern parts would suffer from desertification [5]. This would ultimately influence the mycotoxicological portrait of preharvest and postharvest cereals.

Studies on Swedish and Romanian cereals claim that climatic changes considerably affect the variations in mycotoxin levels [4,6,7].

Mycotoxins are generally considered toxic for humans and animals. Thus, some of them (aflatoxins, ochratoxin A, deoxynivalenol, zearalenone, fumonisins, T-2, HT-2, and ergot alkaloids) are included in the legislative documents set by the European Union (EU) [8,9]. Nevertheless, many of them are still not acknowledged by legislative institutions. These are generally conjugated toxins that transform digestion, food processing, or via plant enzyme systems into a modified form (glucosides, sulfates, acetyl forms, etc.), or the conjugated form is degraded into the original molecule [10]. Some studies confirm that the application of fungicides does not help suppress mycotoxin synthesis in crops; on the contrary, it encourages it. This mainly depends on the climatic conditions, time of fungicide application, and type of fungicide. Hauser Hahn et al. [11] reported that a combination of triazoles (tebuconazole + protioconazole) effectively controls Fusarium head blight and can significantly decrease mycotoxin synthesis. Horky et al. [12] determined that during rainy seasons, fungicide treatment did not help reduce mycotoxins in crops. Rickes da Luz et al. [13] determined that antifungal treatments induced higher concentrations of aflatoxin 1 (AFB1) in wheat in comparison to the control as if it acted as an additional stressor for the plant. Havlova et al. [14] reported similar results and noted that the application of fungicides affects the biochemical mechanisms of grain development, which can result in a modified chemical structure of grain. This can be significant for the end-use industry. Besides the preharvest measures, it is important to apply appropriate postharvest measures and adjust storage conditions to the harvested grain to successfully reduce the occurrence of mycotoxins [15,16].

Some of the most significant mycotoxins to date are deoxynivalenol (DON), zearalenone (ZEA), aflatoxin B1, fumonisins, ochratoxin A, T-2, and HT-2 toxins. They are a result of different fungal genera metabolism (*Fusarium*, *Aspergillus*, *Alternaria*, etc.) [17,18,19]. However, there are over 300 mycotoxins that have been detected so far [20,21], and only a fraction of them are included in the legislation with set limit concentrations. Modified or masked mycotoxins, mycotoxins which undergo biochemical modification during ingestion and then return to their native mycotoxin form, can be hazardous as well. However, among such a huge number of toxic compounds, only a few are designated and their toxicity known. Many of them are still not investigated, or the consequences of their actions on humans are unknown as they are still emerging. Mycotoxins investigated in this research include ones that are known (such as DON) but also ones designated as emerging: culmorin, 15-hydroxyculmorin, 5-hydroxyculmorin, aurofusarin, siccanol, infectopyron, and tryptophol. Detection and quantification of these mycotoxins are not common for cereals, and a small amount of data are available, since it is mostly mycotoxins that are regulated by the European legislation that are studied [22,23,24,25,26,27,28,29].

The undeniable climatic changes combined with antifungal treatments affect the grain quality of small cereals. As mentioned before, the application of fungicides has to be on time, being best at anthesis or just after anthesis. Antifungal treatment 20 days after anthesis reduced mycotoxin concentration in matured grain without reducing FHB (Fusarium head blight) severity. Nevertheless, the application just at the anthesis reduced the FHB occurrence. Besides the effect on familiar mycotoxins such as DON, the application of fungicides can reduce the occurrence of emerging and modified mycotoxins, as reported by Yoshida et al. [30]. It is known that there are different classes of fungicides, and they can act against fungal hyphae or spores. Most common fungicides belong to a triazole group. The usual active ingredients in these groups are epoxiconazole, metconazole, tebuconazole, triadimefon, flusilazole, etc. [31].

This research is a continuation of the previously published paper by Habschied et al. [32]. This research aimed to assess the content of different metabolites in barley treated with two different antifungal agents, one with protiokonazol + tebuconazole as the active compound and the second treated with metconazole. The results were collected over two years. Ten different hulled barley varieties were investigated concerning micro-climatic conditions during growing seasons, and mycotoxins were determined after harvest.

## 2. Materials and Methods

### 2.1. Barley Samples

In this research, samples of ten hulled barley varieties:

Zlatko;

Barun;

Bravo;

Casanova;

Maxim;

Maestro;

Bepo;

Favorit;

Lord;

Oliver;

were collected from trial fields of the Agricultural Institute in Osijek, Osijek (45°32′ N, 18°44′ E). Hulled barley varieties were treated in two different treatments resulting in 10 samples treated with treatment 1 (protiokonazol + tebuconazole; 1 L/ha) and 10 samples treated with treatment 2 (metconazole; 1 L/ha) at heading time. Ten samples were used as control and were not subjected to any treatments. Samples were collected during two consecutive seasons (2019 and 2020). Field experiments were conducted in randomized block designs (RCBDs) with six replications with plot size 7.56 m^2^. Soil properties and climatic conditions during the growing seasons (October–June) can be seen in Table 1. Barley subsamples (1 kg) were taken using a sample spear from a total sample packaged in 10 kg paper bags. Subsamples were taken from the bottom, middle, and top of the bagged barley mass. To obtain a uniform composite sample, the collected subsamples were then mixed in box A and divided into two boxes labeled B and C. Grains from box B were then weighed (200 g) into paper bags. Sampling was performed on cleaned and processed barley grains, and the obtained samples were kept refrigerated in sterile, dry containers. The same procedure was applied to every variety.

May and June are the most important months for mycotoxin production; thus, the weather conditions during flowering (May) and harvest (June) seem to be of most significance for mycotoxin and other metabolite levels in harvested barley seeds. For that reason, Table 2 represents the statistical analysis of temperature and precipitation values in May and June for each year. Data regarding temperature and precipitation were obtained by the Croatian Meteorological and Hydrological Service.

### 2.2. Analysis of Metabolites

In brief, 5 g of the homogenized ground sample (Cyclotec™ 1093; Foss Tecator, Hoeganaes, Sweden; 1 mm mesh size) was extracted with an extraction solvent consisting of acetonitrile–water–acetic acid = 79:20:1 for 90 min by using a GFL 3017 rotary shaker (GFL, Burgwedel, Germany) at 180 rpm and at room temperature. Following extraction, a crude sample was precipitated, and 500 μL of the clear extract was diluted with dilution solvent (acetonitrile–water–acetic acid = 20:79:1). For the separation, the Agilent 1290 UHPLC (Agilent Technologies Santa Clara, CA, USA) system was used combined with a Gemini^®^ C18 (Phenomenex, Torrance, CA, USA) (150 × 4.6-mm i.d., 5 μm particle size) column, and a C18 security guard cartridge, 4 × 3 mm i.d., while the Sciex 5500 QTrap^®^ (Sciex, Toronto, ON, Canada) system was used for detection and quantification. All system parameters were as described in Sulyok et al. [33], and all analyses were performed in triplicate.

### 2.3. Statistical Data Analysis

The results were subjected to analysis of variance (ANOVA) and Fisher’s least significant difference (LSD) test, with significance defined at *p* < 0.05. Statistical analysis was carried out using Statistica 12.7 (StatSoft Inc., Tulsa, OK, USA, 2015).

## 3. Results and Discussion

Weather conditions for both years are described in Table 1 and Table 2. The results of this research are presented in Table 3.

This research was a continuation of the previous research [32] and continued to monitor the occurrence of mycotoxins in barley. However, in this experiment, two treatments were introduced at heading time, the first being a fungicide containing protiokonazol + tebukonazol, while the second contained metconazole as an active compound. The results published by Habschied et al. [32] in 2019 showed significantly lower values for determined mycotoxins than quantified in this research, even though no fungicide was applied. The temperatures were similar to those in the monitored years, but precipitation in May, during the crucial flowering period, was significantly higher in 2019 than in 2016/2017/2018. The most significant mycotoxin determined in the samples seems to be DON. DON has been introduced into legislation and is considered one of the most widespread mycotoxins. It can be found in different cereals and despite different agro-technical and climatic conditions. However, DON, culmorin, 15-hydroxyculmorin, 5-hydroxyculmorin, and aurofusarin were not detected in 2020, an arid year, but three of the emerging mycotoxins, siccanol, infectopyron, and unspecific metabolite tryptophol, showed consistency in occurrence for both years and regardless of the treatment.

In 2019, the difference between the treatments is significant, especially for DON. DON was detected in all samples in 2019. However, some varieties showed greater resistance toward DON contamination, regardless of the treatment. Bravo, Favorit, and Oliver showed higher resistance toward DON contamination when not subjected to any treatment. The control samples had the lowest concentrations of DON in comparison to the samples treated with chosen fungicides. For Bravo, this was the case for all the mycotoxins; they were all lower in the control sample than in the treated samples. The increase in DON was significant in treatment 2 (1809 µg/kg) in relation to the control sample (196 µg/kg), reaching almost 90% for the Favorit variety. A somewhat lower increase was detected in Bravo, where it reached −85% of DON in the control sample regarding treatment 1. The Oliver variety had the lowest reduction rate in the control sample, where it amounted to ca. 48% in comparison to treatment 1, which is still a significant number.

Favorit had a significantly higher concentration of DON in samples treated with treatment 2 (1809 µg/kg) than in the control or treatment 1 in the year 2019. It is interesting that here too the lowest concentration of DON was detected in the control sample (195 µg/kg).

The same trend can be noted for culmorin, 15-hydroxyculmorin, and 5-hydroxyculmorin. Siccanol and infectopyron were suppressed and showed lowest concentrations in 2019 in samples treated with treatment 2.

DON levels are usually good predictors of DON conjugates in commodities [32,34]. Similarly, it can be noted in this research that *Fusarium* mycotoxins such as 15-hydroxyculmorin, 5-hydroxyculmorin, and aurofusarin could be correlated with DON concentrations for each sample. The only *Fusarium* toxin that showed randomness in this sense is siccanol. Its concentrations do not follow the DON concentrations, as with other *Fusarium* toxins. The highest DON level was detected in the Maestro sample treated with treatment 2, and it amounted to 2658 µg/kg. This is well beyond, more than ten-fold higher, the prescribed legislative limit. Besides DON, culmorin (1393 µg/kg), 15-hydroxyculmorin (2626 µg/kg), 5-hydroxyculmorin (3736 µg/kg), and aurofusarin (443 µg/kg) also exhibited the highest levels in this sample quantified in this research.

Siccanol, infectopyron, and tryptophol showed higher concentrations in samples subjected to treatment 1 than in the control samples and treatment 2 in 2019. Siccanol is a *Fusarium* metabolite, while infectopyron originates from the genus *Alternaria*. This implies that both species were abundantly present in 2019 in the fields. This is not surprising since the weather conditions in 2019, especially in May and June, during flowering and harvest season, held a lot of rain (Table 2). The precipitation in May reached 150.8 mm, and the temperature was 14.0 °C. This is significantly higher precipitation than in 2020 (112.8 mm), followed by a lower mean temperature (15.3 °C) during the flowering period. *Fusarium* species thrive in such conditions, hence the high DON, culmorin, 15-hydroxyculmorin, 5-hydroxyculmorin, and aurofusarin levels in all samples in 2019, regardless of which treatment was applied. Treatment 2 seems to be efficient in suppressing siccanol, infectopyrin, and tryptophol in samples of the Favorit variety. Despite obviously being present in the field in 2020, *Fusarium* and *Alternaria* spp. did not synthesize as many mycotoxins as in 2020. Namely, DON, culmorin, 15-hydroxyculmorin, 5-hydroxyculmorin, and aurofusarin were not detected. However, siccanol, infectopyrin, and tryptophol were produced in somewhat lower concentrations, but still detectable.

Oliver showed different results regarding mycotoxin concentrations in relation to the treatment. Namely, Oliver showed lower values for all mycotoxins subjected to treatment 2, except for culmorin and siccanol. DON, 15-hydroxyculmorin, 5-hydroxyculmorin, aurofusarin, infectopyrin, and tryptophol all showed lower values when treated with treatment 1 than when treatment 2 was applied. It seems that Oliver responds better to treatment 1 when it comes to *Fusarium* mycotoxins.

In 2019, most of the samples contained extremely high levels of mycotoxins, levels which are significantly higher than the European Union legislation allows in cereals.

As 2020 was a dry and warm year, there were not detectable concentrations of DON, culmorin, 15-hydroxyculmorin, or 5-hydroxyculmorin. No matter the treatment, or no treatment (control), none of the varieties contained detectable amounts of DON, culmorin, 15-hydroxyculmorin, or 5-hydroxyculmorin. Namely, in 2020, only siccanol, infectopyron, and tryptophol showed detectable concentrations in almost all samples. There are significant differences between the samples this year; however, it appears that fungicide application was more effective in suppressing mycotoxin synthesis than in 2019. Namely, the control samples contained the highest amount of mycotoxins in comparison to the treated samples. It appears that both treatments acted efficiently against DON, culmorin, 15-hydroxyculmorin, and 5-hydroxyculmorin. They acted less effectively against siccanol, infectopyron, and tryptophol in most samples. Oliver, Lord, Maestro, Maxim, Barun, and Zlatko showed the lowest values for siccanol, infectopyron, and tryptophol when treated with treatment 2. Bravo appeared to be again the most resistant to infection since, in 2020, its control showed the lowest values for siccanol (<LOD—level of detection) in comparison to the samples treated with both treatments. Zlatko was the only variety that showed low concentrations of *Fusarium* mycotoxins in 2020. They were detected in a Zlatko sample treated with treatment 1. Even though they were detected in low concentrations, DON, culmorin, and 15-hydroxyculmorin were successfully quantified.

The most interesting finding obtained from this research is the fact that no matter what fungicide is applied during rainy years, mycotoxins cannot be suppressed sufficiently. Another interesting finding was that infectopyron and siccanol occurred in both years, no matter the treatment. This could imply that these two mycotoxins could be a new problem for crop growers and the processing industry. Namely, they were detected in all samples in 2019 and in most of the samples in 2020. Penagos-Tabares et al. [17] studied mixtures of mycotoxins, phytoestrogens, and pesticides co-occurring in wet spent brewery grains, and they noted a 100% occurrence of infectopyron and siccanol in samples. Infectopyron had the highest average and maximum concentration of all *Alternaria* spp. metabolites, and siccanol of *Fusarium* metabolites. They noted that these are mycotoxins with unfamiliar biological activities. This makes them potentially hazardous for humans and animals and should be further explored, as reported by Andersen et al. [35] and Larsen et al. [36]. The highest concentration of siccanol in this research was 1260 µg/kg in a sample protected with treatment 2 (Lord in 2019), while in research conducted by Penagos-Tabares et al. [17], siccanol reached a concentration of 966 µg/kg in brewer’s grain. These are significantly high concentrations. Concentrations of infectopyron in 2020 were significantly lower than in 2019, and some varieties showed greater resistance in 2020, which is greatly related to the fungicidal treatment. The highest level of infectopyron was detected in 2019 in the Maestro, at 536 µg/kg. However, Bravo showed the lowest concentrations in 2020, with control samples being free from both mycotoxins, siccanol and infectopyron. This is similar to a study Kleber et al. [15] published in 2023. Namely, in this research infectopyron showed an increase after the antifungal treatment [15]. Combination of infectopyron + siccanol in almost all samples indicated that they have a strong coincidence and should be monitored together, similarly to DON+T2/HT2 [37]. This research can highlight that siccanol, a *Fusarium* metabolite, co-occurs with infectopyron, an *Alternaria* mycotoxin.

This indicates a further conclusion: that *Alternaria* and *Fusarium* spp. successfully co-occur in today’s climatic conditions. And even though DON or other *Fusarium* mycotoxins are not detectable, siccanol can be quantified in almost all samples. It seems that antifungal treatments do not suppress infectopyron and siccanol even in arid years. However, this does not apply to DON and other *Fusarium* mycotoxins; they were not even detected in the samples in 2020.

The occurrence of tryptophol showed variations in concentrations, but not as big between the years as other metabolites. The highest concentration was quantified in 2019 in the Zlatko variety, treated with fungicide 1. Tryptophol is an aromatic alcohol formed as the end product of tryptophan catabolism [38]. On average, barley kernels contain 0.7% tryptophan by dry weight [39]. Since tryptophol is classified as an unspecific metabolite, it is probably synthesized by the plant itself [32]. Namely, it acts like plant hormones and is synthesized when a plant finds itself in favorable conditions. Landry and Delhaye [40] reported that tryptophan content is linearly related to the nitrogen content in grains, and Knežević et al. [41] published a study where they detected tryptophan in one barley cultivar and all six analyzed wheat lines. This supports the conclusion that tryptophol is a result of tryptophan metabolism in plants. Even though 2019 was rainy and as a result, significant amounts of metabolite were detected, it seems that it was more favorable for barley growth, as more tryptophol was produced. This is not surprising since rain encourages plant growth, especially since the agrotechnical measures of fertilization in the field were the same for all samples (control, treatment 1, and treatment 2). In 2020, the concentrations of this compound seem random; for some varieties, this compound was not detected. In Barun, Bravo, Maxim, and Maestro, treatment 2 was particularly successful in suppressing the synthesis of this metabolite.

Even though the application of fungicides has been proven to reduce mycotoxin concentrations [15], widespread climatic changes dictate the results of antifungal treatments. Namely, certain years undergo such dramatic changes in temperature and precipitation amount that it is impossible to predict which treatment should be applied for a specific crop, or, as it would be better to say, variety. In any case, this could pose an emerging challenge for the malting and brewing industry. Namely, future research should examine the behavior of emerging mycotoxins, such as siccanol and infectopyron, and other metabolites such as tryptophol, during malting and brewing. This could potentially cause serious problems in both industries, especially as these metabolites are occurring no matter the treatment. Another required action is the follow-up on the transfer from barley to malt to beer. Finally, finding an efficient suppressant in the form of a pesticide would help protect the barley quality, malting, and brewing industries, and consequently the consumers from potentially harmful effects.

## 4. Conclusions

Mycotoxins have been identified as major fungal metabolites that have adverse effects on human health. Monitoring and regulation have been established in all parts of the world. Known mycotoxins have been controlled via different fungicides, and every batch with an excess of prescribed concentrations for DON or any other regulated mycotoxins is withdrawn from the market. There are, however, unregulated, and emerging mycotoxins that have not been introduced into legislation and are still occurring in different amounts in cereals. This research has established that siccanol and infectopyron are among the commonly occurring mycotoxins in barley, especially during rainy seasons. It is notable that 2019 was a particularly wet year with lots of rain periods during the flowering season in May. It was expected that mycotoxins such as DON would be found in significant amounts. However, it seems that the application of fungicides did not help in suppressing the occurrence of mycotoxins; in some cases, it even resulted in higher amounts of mycotoxins. This indicates that fungicides can act as additional stressors for plants. To conclude, suppression of mycotoxin production during rainy years is not efficient, but further studies should be conducted to confirm the results obtained in this study; however, it points to the need to adjust the treatments, whether to reschedule them or to combine several fungicides since they do not work in rainy years when applied separately. A relation between *Fusarium* and *Alternaria* toxins and climatic conditions has been established in this research, and it shows that infectopyron has been identified as a major *Alternaria* toxin for both years. Is there an urgent need for toxicological data for infectopyron?

## Figures and Tables

**Table 1:** Precipitation and mean temperature during the growing season of winter barley (October–June).

Season	Mean Precipitation (mm)	Total Precipitation (mm)	Mean Temperature (°C)
2018/19	52.66	473.90	9.6
2019/20	40.96	369.60	9.9

**Table 2:** Meteorological analysis of the temperature and precipitation in May and June for each year.

	2019	2020
**Temperature (°C)**		
May	14.0	15.3
June	23.1	20.2
**Precipitation (mm)**		
May	150.8	112.8
June	53.3	73.5

**Table 3:** Detected mycotoxins.

Variety/Tretament/Year	Deoxynivalenol	Culmorin	15-Hydroxyculmorin	5-Hydroxyculmorin	Aurofusarin	Siccanol	Infectopyron	Tryptophol
				µg/kg				
ZLATKO_2019_1	278 ^b^	205 ^c^	345 ^b^	313 ^b^	6.26 ^b^	153 ^d^	51.8 ^e^	20.5 ^d^
ZLATKO_2019_2	256 ^c^	222 ^a^	94.3 ^c^	<LOQ	<LOD	<LOD	<LOQ	102 ^a^
ZLATKO_2019_3	447 ^a^	219 ^b^	370 ^a^	415 ^a^	177 ^a^	793 ^a^	327 ^a^	25.9 ^c^
ZLATKO_2020_1	<LOD	<LOD	<LOD	<LOD	<LOD	182 ^c^	129 ^d^	19.9 ^d^
ZLATKO_2020_2	<LOD	<LOD	<LOD	<LOD	<LOD	234 ^b^	212 ^b^	39.6 ^b^
ZLATKO_2020_3	<LOD	<LOD	<LOD	<LOD	<LOD	139 ^e^	206 ^c^	25.5 ^c^
BARUN_2019_1	1046 ^a^	737 ^a^	810 ^a^	1237 ^a^	200 ^a^	1242 ^a^	313 ^a^	38.6 ^a^
BARUN_2019_2	245 ^c^	176 ^c^	174 ^c^	84.6 ^c^	15.2 ^c^	224 ^c^	47.9 ^e^	31.9 ^c^
BARUN_2019_3	873 ^b^	443 ^b^	657 ^b^	922 ^b^	190 ^b^	753 ^b^	238 ^b^	32.7 ^b^
BARUN_2020_1	<LOD	<LOD	<LOD	<LOD	<LOD	166 ^e^	195 ^c^	21.4 ^d^
BARUN_2020_2	<LOD	<LOD	<LOD	<LOD	<LOD	170 ^d^	148 ^d^	14.7 ^e^
BARUN_2020_3	<LOD	<LOD	<LOD	<LOD	<LOD	<LOD	<LOD	<LOD
BRAV0_2019_1	131 ^c^	75.7 ^c^	116 ^c^	202 ^c^	6.39 ^c^	159 ^c^	57.6 ^d^	28.4 ^c^
BRAV0_2019_2	853 ^a^	483 ^b^	670 ^a^	697 ^b^	111 ^a^	816 ^a^	423 ^a^	42.5 ^b^
BRAV0_2019_3	809 ^b^	542 ^a^	497 ^b^	811 ^a^	93.7 ^b^	692 ^b^	250 ^b^	52.6 ^a^
BRAV0_2020_1	<LOD	<LOD	<LOD	<LOD	<LOD	<LOD	114 ^c^	19.3 ^d^
BRAV0_2020_2	<LOD	<LOD	<LOD	<LOD	<LOD	77.8 ^e^	<LOQ	15.6 ^e^
BRAV0_2020_3	<LOD	<LOD	<LOD	<LOD	<LOD	89.1 ^d^	<LOQ	<LOQ
CASANOVA_2019_1	1567 ^a^	691 ^a^	935 ^a^	1455 ^a^	195 ^a^	763 ^b^	316 ^b^	26.7 ^c^
CASANOVA_2019_2	648 ^c^	251 ^c^	580 ^b^	810 ^b^	164 ^b^	649 ^c^	202 ^e^	33.0 ^a^
CASANOVA_2019_3	791 ^b^	394 ^b^	437 ^c^	640 ^c^	39.6 ^c^	1019 ^a^	386 ^a^	31.9 ^b^
CASANOVA_2020_1	<LOD	<LOD	<LOD	<LOD	<LOD	171 ^d^	297 ^c^	16.6 ^e^
CASANOVA_2020_2	<LOD	<LOD	<LOD	<LOD	<LOD	87 ^f^	63.6 ^f^	20.8 ^d^
CASANOVA_2020_3	<LOD	<LOD	<LOD	<LOD	<LOD	148 ^e^	273 ^d^	14.7 ^f^
MAXIM_2019_1	570 ^b^	338 ^b^	344 ^b^	516 ^b^	160 ^b^	694 ^c^	306 ^c^	29.7 ^b^
MAXIM_2019_2	432 ^c^	282 ^c^	279 ^c^	292 ^c^	196 ^a^	977 ^a^	343 ^b^	24.7 ^c^
MAXIM_2019_3	1518 ^a^	619 ^a^	837 ^a^	990 ^a^	47.6 ^c^	798 ^b^	358 ^a^	22.0 ^d^
MAXIM_2020_1	<LOD	<LOD	<LOD	<LOD	<LOD	177 ^d^	190 ^d^	<LOD
MAXIM_2020_2	<LOD	<LOD	<LOD	<LOD	<LOD	161 ^e^	170 ^e^	30.9 ^a^
MAXIM_2020_3	<LOD	<LOD	<LOD	<LOD	<LOD	59.5 ^f^	<LOQ	<LOD
MAESTRO_2019_1	1603 ^b^	1071 ^b^	1024 ^b^	516 ^b^	320 ^b^	618 ^c^	267 ^d^	18.9 ^d^
MAESTRO_2019_2	1159 ^c^	262 ^c^	845 ^c^	292 ^c^	165 ^c^	901 ^b^	536 ^b^	52.0 ^a^
MAESTRO_2019_3	2659 ^a^	1393 ^a^	2627 ^a^	990 ^a^	444 ^a^	1054 ^a^	401 ^a^	25.3 ^c^
MAESTRO_2020_1	<LOD	<LOD	<LOD	<LOD	<LOD	222 ^d^	350 ^c^	30.0 ^b^
MAESTRO_2020_2	<LOD	<LOD	<LOD	<LOD	<LOD	144 ^e^	114 ^e^	18.6 ^d^
MAESTRO_2020_3	<LOD	<LOD	<LOD	<LOD	<LOD	<LOQ	<LOQ	<LOQ
BEPO_2019_1	1444 ^a^	645 ^a^	973 ^a^	1284 ^a^	142 ^c^	764 ^b^	242 ^c^	25.7 ^d^
BEPO_2019_2	1231 ^b^	589 ^b^	732 ^b^	1105 ^b^	172 ^a^	760 ^c^	244 ^b^	27.9 ^c^
BEPO_2019_3	835 ^c^	414 ^c^	492 ^c^	554 ^c^	167 ^b^	936 ^a^	235 ^d^	22.1 ^e^
BEPO_2020_1	<LOD	<LOD	<LOD	<LOD	<LOD	137 ^e^	287 ^a^	33.7 ^b^
BEPO_2020_2	<LOD	<LOD	<LOD	<LOD	<LOD	<LOQ	128 ^e^	42.2 ^a^
BEPO_2020_3	<LOD	<LOD	<LOD	<LOD	<LOD	161 ^d^	129 ^e^	25.1 ^d^
FAVORIT_2019_1	196 ^c^	107 ^c^	128 ^c^	71.8 ^c^	331 ^a^	876 ^b^	265 ^b^	21.2 ^d^
FAVORIT_2019_2	867 ^b^	483 ^b^	667 ^b^	853b	148 ^c^	921 ^a^	295 ^a^	37.6 ^a^
FAVORIT_2019_3	1809 ^a^	1357 ^a^	932 ^a^	1094 ^a^	279 ^b^	655 ^c^	213 ^c^	25.6 ^c^
FAVORIT_2020_1	<LOD	<LOD	<LOD	<LOD	<LOD	103 ^e^	164 ^d^	19.9 ^e^
FAVORIT_2020_2	<LOD	<LOD	<LOD	<LOD	<LOD	<LOQ	141 ^e^	27.6 ^b^
FAVORIT_2020_3	<LOD	<LOD	<LOD	<LOD	<LOD	129 ^d^	118 ^f^	19.2 ^e^
LORD_2019_1	2134 ^a^	486 ^b^	1946 ^a^	1715 ^b^	341 ^a^	625 ^c^	409 ^a^	31.9 ^a^
LORD_2019_2	837 ^c^	368 ^c^	838 ^c^	1008 ^c^	314 ^b^	934 ^b^	218 ^e^	26.9 ^c^
LORD_2019_3	1667 ^b^	500 ^a^	1278 ^b^	1742 ^a^	144 ^c^	1261 ^a^	348 ^b^	25.2 ^d^
LORD_2020_1	<LOD	<LOD	<LOD	<LOD	<LOD	110 ^f^	68.4 ^f^	20.9 ^f^
LORD_2020_2	<LOD	<LOD	<LOD	<LOD	<LOD	137 ^e^	265 ^c^	22.8 ^e^
LORD_2020_3	<LOD	<LOD	<LOD	<LOD	<LOD	312 ^d^	250 ^d^	30.5 ^b^
OLIVER_2019_1	771 ^c^	312 ^c^	619 ^c^	637 ^c^	140 ^b^	7220 ^b^	217 ^d^	44.3 ^b^
OLIVER_2019_2	1469 ^a^	318 ^b^	1047 ^a^	1104 ^a^	282 ^a^	530 ^c^	492 ^a^	36.7 ^c^
OLIVER_2019_3	927 ^b^	417 ^a^	722 ^b^	776 ^b^	95.6 ^c^	7610 ^a^	250 ^c^	21.2 ^d^
OLIVER_2020_1	<LOD	<LOD	<LOD	<LOD	<LOD	106 ^d^	298 ^b^	20.2 ^d^
OLIVER_2020_2	<LOD	<LOD	<LOD	<LOD	<LOD	58.8 ^e^	121 ^e^	20.4 ^d^
OLIVER_2020_2	<LOD	<LOD	<LOD	<LOD	<LOD	58.8 ^e^	118 ^f^	48.67 ^a^

1—control; 2—protiokonazol + tebukonazol; 3—metconazole. Means within columns with different superscripts are significantly different (*p* < 0.05). LOD—limit of detection.

## Data Availability

Data are available upon request from the corresponding author.
